# Clinicopathological and prognostic significance of zinc finger antisense 1 overexpression in cancers: A meta-analysis

**DOI:** 10.1097/MD.0000000000013378

**Published:** 2018-12-10

**Authors:** Yuanxiu Leng, Qing Luo, Xumei Chen, Fang Chen, Xue Wang, Yana Pan

**Affiliations:** a Department of Oncology Laboratory, Cancer Hospital, Affiliated Hospital of Zunyi Medical University, Zunyi, Guizhou Province, Zunyi; b Hematology and Oncology Department, The Fifth Affiliated Hospital of Zunyi Medical University, Zhuhai, Guangdong Province, Doumen District, Zhuhai,China.

**Keywords:** cancer, long noncoding RNA, meta-analysis, ZFAS1

## Abstract

**Background:**

An increasing number of studies have recently highlighted the role of zinc finger antisense 1(ZFAS1) as a prognostic marker in cancers. However, these results remain controversial. Hence, a meta-analysis was conducted to further investigate the effects of ZFAS1 expression on clinicopathological features and survival outcomes.

**Method:**

All eligible studies were searched from PubMed, Embase, Web of Science, and the Cochrane Library. All included articles evaluated the relationship between the expression levels of ZFAS1 and survival, or the range of pathological features in cancer patients. Pooled hazard ratios (HRs) with 95% confidence intervals (CIs) were computed to evaluate the effect of ZFAS1 expression on overall survival (OS), relapse-free survival (RFS), and disease-free survival (DFS). The relationship between ZFAS1 expression and clinicopathological features was determined through pooled odds ratios (ORs) and 95% CIs.

**Results:**

In total 8 studies, which comprised of 820 patients, were qualified for analysis. Results revealed that the overexpression of ZFAS1 was significantly associated with poor OS *(*HR = 1.97, 95% CI: 1.53–2.54), worse RFS (HR = 1.95, 95% CI: 1.24–3.04) and worse DFS (HR = 2.35, 95% CI: 1.43–3.88) in cancers. Further subgroup analysis revealed that ZFAS1 overexpression was significantly correlated with poor OS in different cancer types, HR obtain methods and sample sizes. In addition, this meta-analysis revealed that the upregulated expression of ZFAS1 was significantly associated with lymph node metastasis, Tumor Node Metastasis (TNM) stage, and tumor size.

**Conclusions:**

This meta-analysis revealed that the expression of ZFAS1 was associated with tumor prognosis. ZFAS1 could be used as a predictor for tumor progression in various cancers.

## 1 Introduction

Long noncoding RNAs (lncRNAs) are a large class of RNA molecules that have a length of more than 200 nucleotides which lacks an open reading frame of significant length (less than 100 amino acids).^[1]^ lncRNAs have drawn particular attentions for their involvements in tumor development especially when dysregulated, which therefore showed great potential as key diagnostic biomarkers.^[2]^ Zhang et al found that the upregulation of lncRNA MALAT1 was correlated with tumor progression and poor prognosis in clear cell renal cell carcinoma.^[3]^ Furthermore, the overexpression of H19 was reported to be associated with poor prognosis in bladder and gastric cancers^[4,5]^. Moreover, the downregulation of lncRNA XIST and JPX has been associated with hepatocellular carcinoma prognosis.^[6]^

Zinc finger antisense 1 (ZFAS1), also known as ZNFX1 antisense RNA1 or ZNFX1-AS1, exhibited a 10-fold decrease in RNA level between pregnancy and lactation. The ZFAS1 locus is host to 3 C/D-box snoRNAs and its transcription initiates from the antisense strand near the 5′ end of the protein-coding gene ZNFX1.^[7]^ It was originally identified as a tumor suppressor gene in human breast^[7]^ and liver cancers.^[8]^ However, in recent years, increasing evidences have demonstrated that lncRNA ZFAS1 was upregulated in various cancers such as colorectal cancer,^[9]^ glioma^[10]^ and gastric cancer.^[11]^ The upregulation of ZFAS1 may promote tumor cell proliferation, invasion, and metastasis. This suggests that ZFAS1 may act as an oncogene in tumorigenicity.^[9,12]^

The clinical studies showing that the upregulation of ZFAS1 expression is associated with poor prognosis in different types of cancer. In addition, ZFAS1 is significantly associated with certain clinicopathological features in different human tumors, such as lymph node metastasis, TNM stage, and tumor size.^[10,12–14]^ However, due to the limited sample size and single center design of the present studies, a systematic review of literature was performed to evaluate and predict the overall risk of high ZFAS1 expression for the survival prognosis and clinicopathological features of patients with various types of cancer.

## 2 Materials and methods

### 2.1 Literature search

PubMed, EMBASE, Web of Science, and the Cochrane Library were systematically searched for comprehensive literature. The last search time was on June 20, 2017. The search strategy included the following terms: “ZFAS1”, “ZXFX1 antisense RNA1”, “ZNFX1-AS1”, and “cancer”, “tumor”, “neoplasm”, “carcinoma”. No language restrictions are used. In addition, relevant literature were manually reviewed to obtain more research results.

### 2.2 Inclusion and exclusion criteria

Inclusion criteria:

(1) studies that have shown the prognostic role of ZFAS1 in patients with different types of cancer;
(2) studies that divided patients into high and low expression groups according to the expression level of ZFAS1;
(3) studies that provided sufficient information to estimate the hazard ratio (HR) or odds ratio (OR), and its 95% confidence intervals (CIs);
(4) studies that identified the relationship between ZFAS1 expression and overall survival (OS), disease-free survival (DFS), recurrence-free survival (RFS), or clinicopathological parameters.

Exclusion criteria:

(1) studies with overlapping or duplicated data;
(2) letters, case reports, reviews, and animal studies;
(3) studies that did not provide sufficient survival data;
(4) non-English studies.

### 2.3 Data extraction and quality assessment

Two researchers (YXL and XMC) independently extracted relevant information based on the inclusion and exclusion criteria. Any disagreements were resolved through discussion with a third investigator (XW). The following information and data were extracted from each study: first author's surname, year of publication, cancer type, number of patients, outcome measures, cut-off value of ZFAS1 expression, HR obtain method, pathology (such as gender, tumor differentiation, lymph node metastasis, tumor size, and TNM stage), and the HRs and 95% CIs of the expression of ZFAS1 for OS, DFS, and RFS.

In order to ensure the quality of the study, 2 researchers (YXL and YMC) independently assessed the quality of included literature using the Newcastle–Ottawa Scale (NOS) (Table 1). It included follow scoring items: 
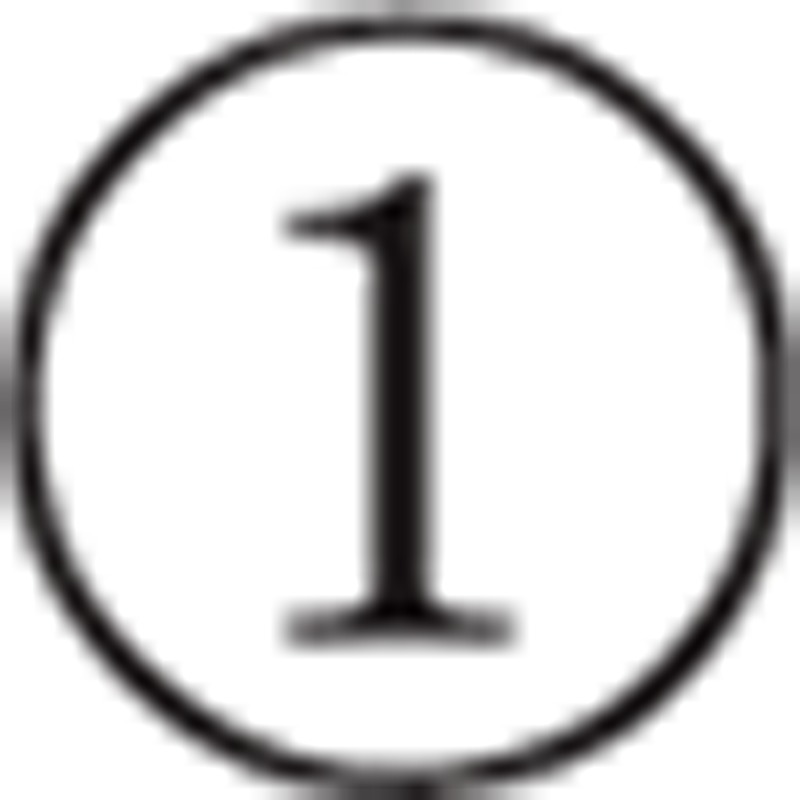
 the representative of the exposed group; 
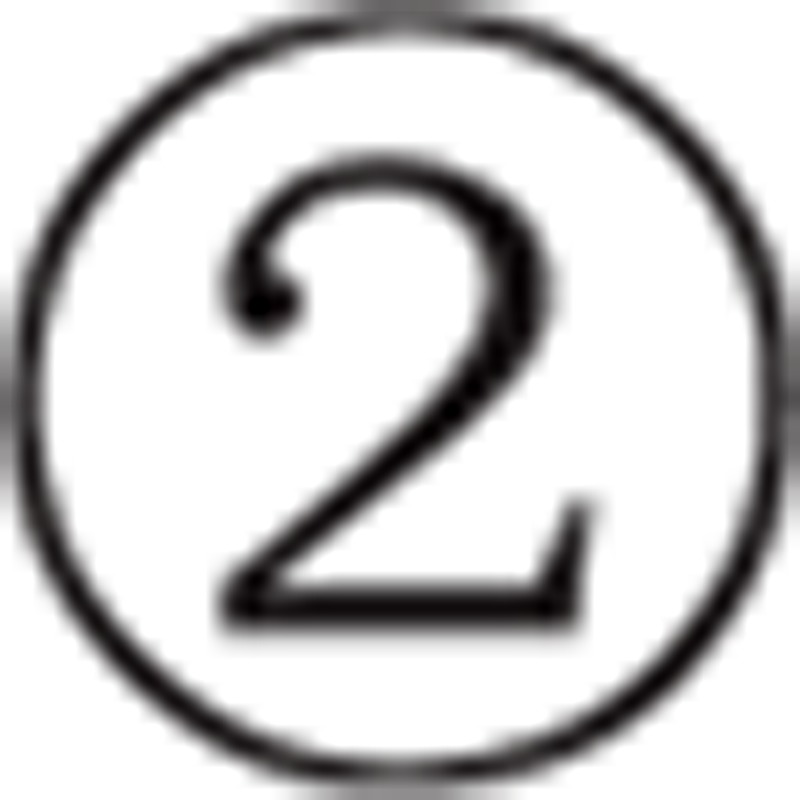
 the choice of the non exposed group; 
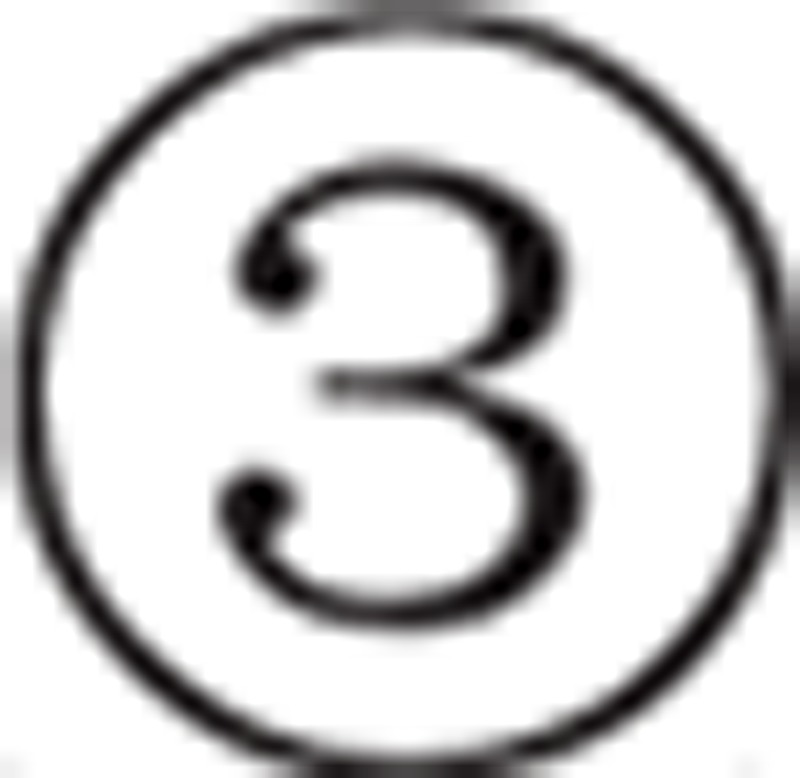
 determination of exposure group; 
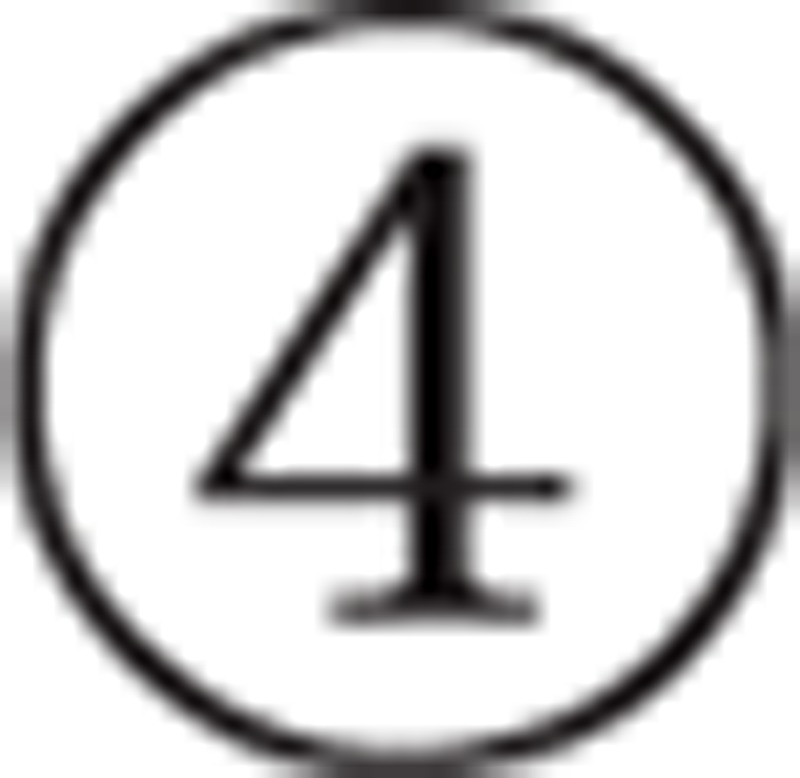
 no study participants had an outcome event before the start of the study; 
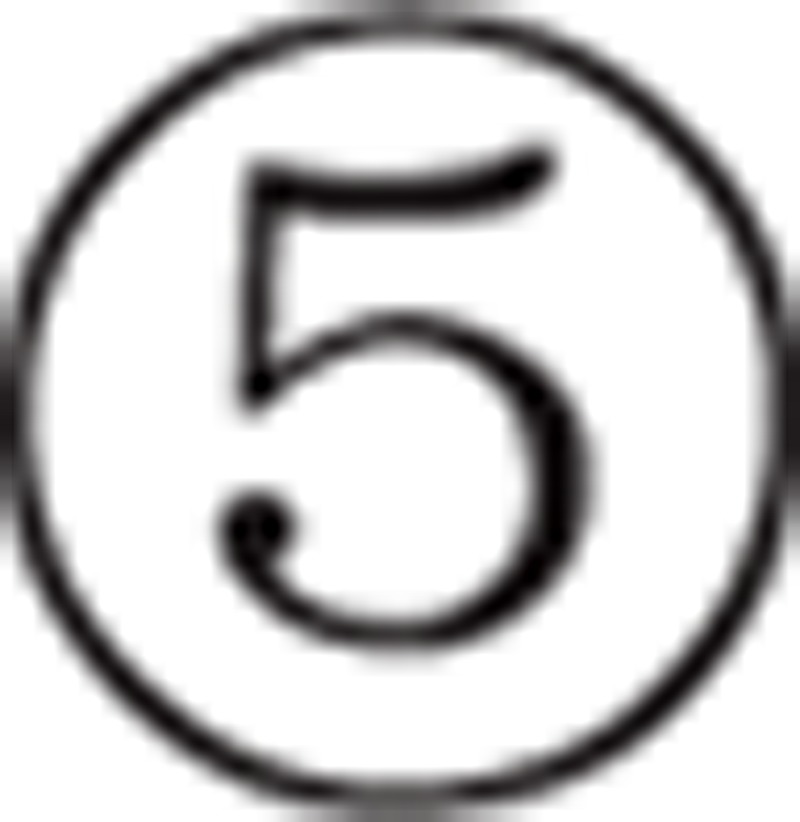
 the comparability of the design or analysis of the study; 
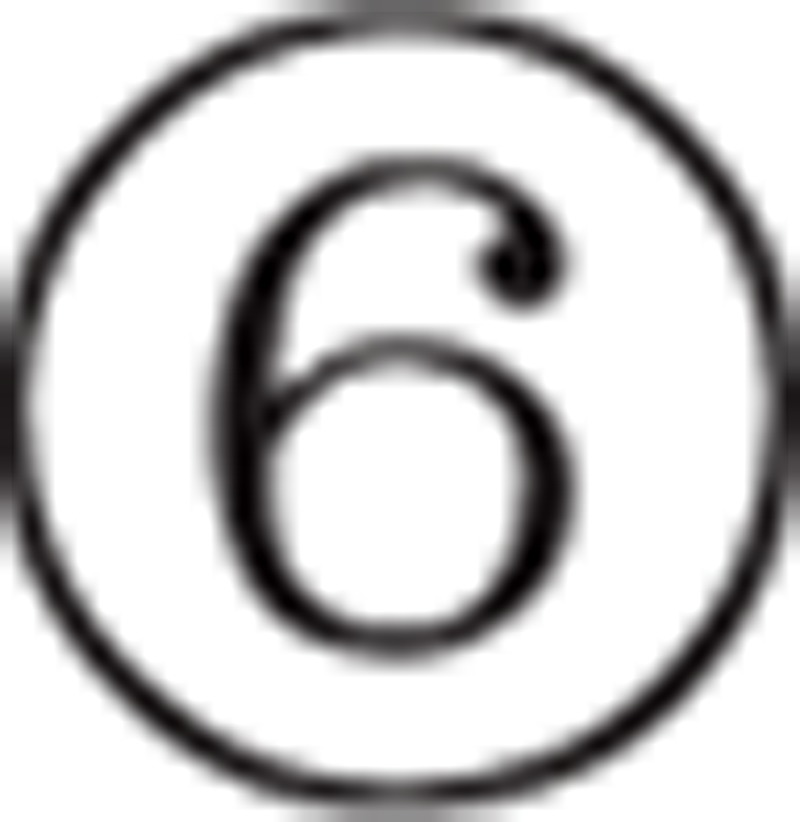
 assessment of outcome events; 
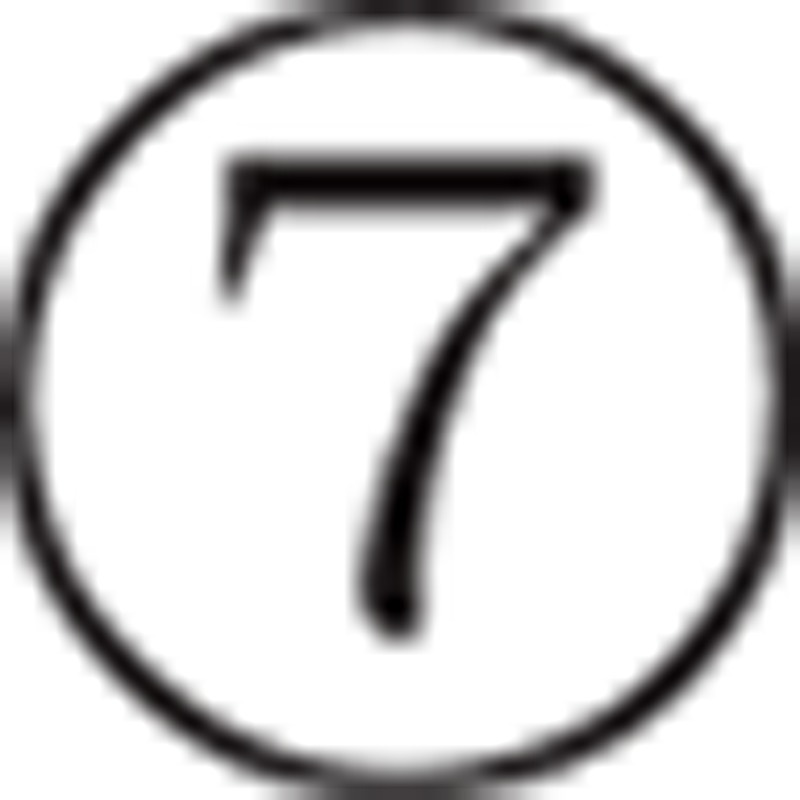
 whether the follow-up time is sufficient; 
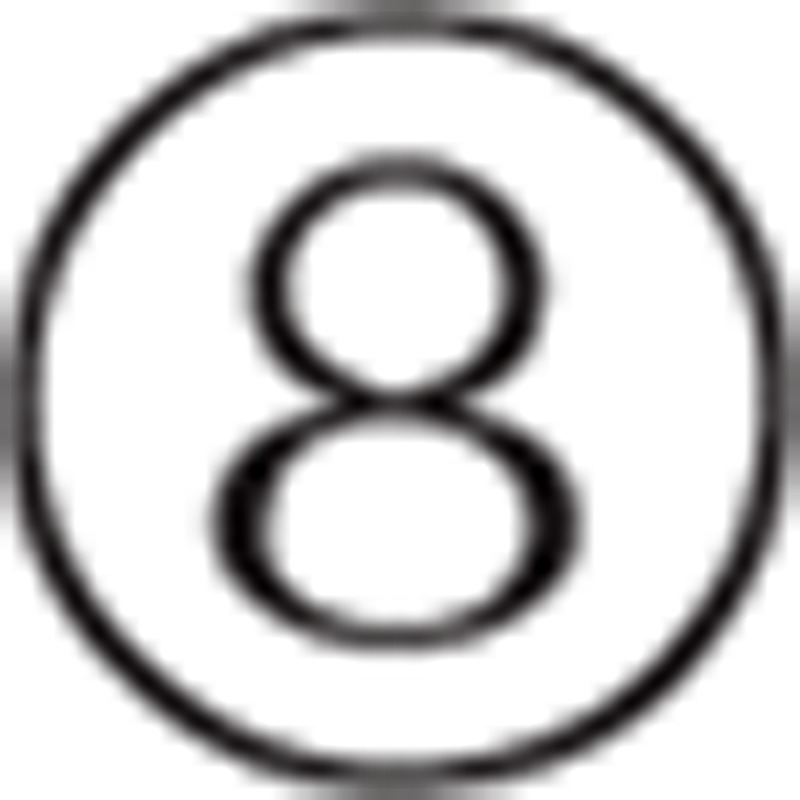
 the integrity of follow up; Fifth accounted for 2 points, the remaining accounted for 1 point, the total score of 9 points. A NOS score ≥5 was considered to be of high quality.

**Table 1:**
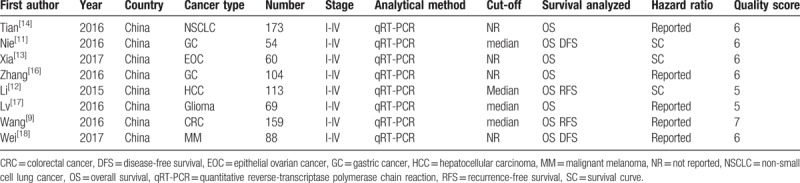
Main characteristics of all included studies.

### 2.4 Statistical methods

Hazard Ratios (HRs) and 95% CIs were calculated for each study. In most studies, HR and 95% CI for OS, DFS, and RFS were directly obtained from the published literature. If these results were obtained by univariate and multivariate analyses, the multivariate analysis was considered to be before the univariate analysis. For studies where HR (95% CI) was not available, data were extracted from the survival curves using Engauge Digitizer version 4.2, and Tierney's method was used to calculate the HRs and 95% CIs.^[15]^ The association between the expression levels of ZFAS1 and the prognosis in various cancers was based on the pooled HRs with 95% CIs. Pooled HR >1 with 95% CI exceeding 1 indicated poor prognosis for the groups with elevated ZFAS1 expression. ORs and 95% CIs were used to evaluate the relationship between ZFAS1 and clinicopathological features, including the gender (male vs female), tumor size (≤5 cm vs >5 cm), differentiation (poorly vs well and moderately), lymph node metastasis (yes vs no), TNM stage (III+IV vs I+II).

Statistical heterogeneity between studies was assessed using the *X2*-based Cochran *Q* test and Higgins *I2*-statistic. A random-effect model was used when the chi-square test was *P* <.10 and the *I*^*2*^*-*statistic values were >50%. Otherwise, a fixed-effect model was used. Subgroup analyses were performed to further dissect the heterogeneity through the HR obtain method, sample size, and cancer type. In addition, publication bias was assessed using Begg funnel plots and Egger linear regression test. All statistical analyses were performed using the software STATA (version 12.0), and the results were considered statistically significant at *P* <.05.

## 3 Results

### 3.1 Basic information of the included studies

**Figure 1:**
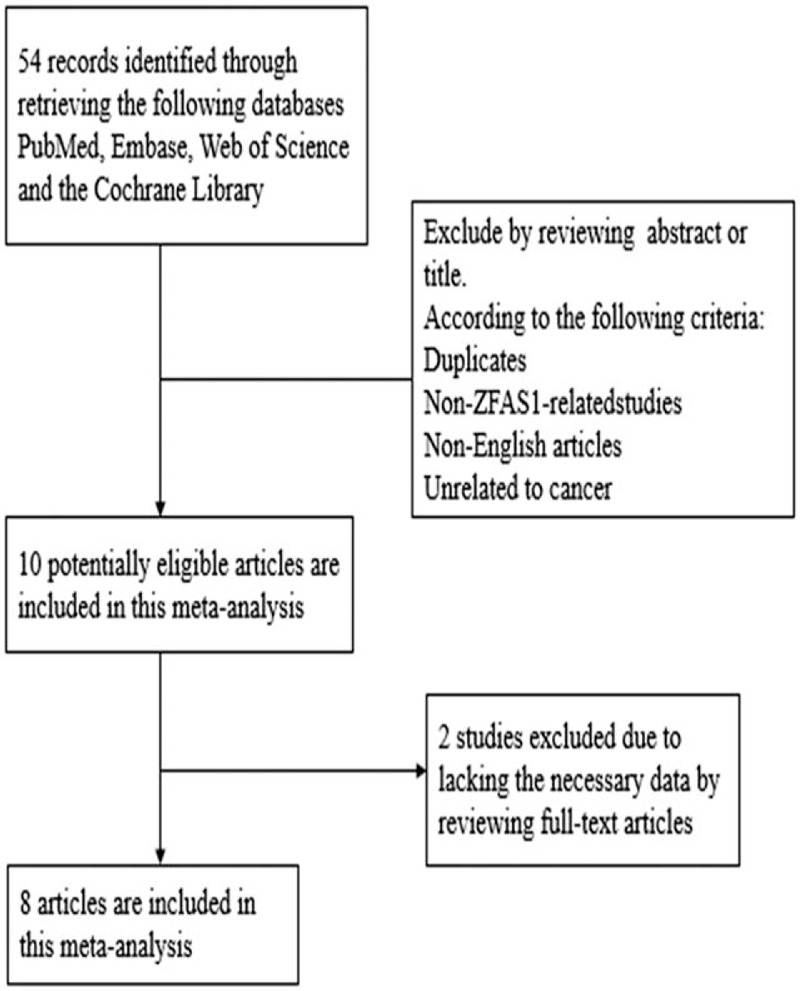
Flow chart of the study selection process and specific reasons for exclusion in the meta-analysis.

In total 54 articles were identified from PubMed, Medline, Web of Science and the Cochrane Library after discreet review (Fig. 1). Among them, 44 articles were excluded due to the following reasons: duplicate studies, non-ZFAS1-related studies, non-English articles or unrelated to cancer. The remaining 10 studies were further evaluated and 2 of which were excluded due to lack of necessary data on the association of ZFAS1 expression with prognosis. Finally, 8 studies were found to be eligible therefore were included in the meta-analysis (Fig. 1).

The main results of the included studies are shown in Table 1. These eligible studies were published between 2015 and 2017 and including a total of 820 patients, with an average number of 102.5 patients per study. The patients in these studies were all Chinese and 7 types of cancers were recorded: 2 gastric cancers (GCs), 1 non-small cell lung cancer (NSCLC), 1 hepatocellular carcinoma (HCC), 1 colorectal cancer (CRC), 1 glioma, 1 malignant melanoma (MM) and 1 epithelial ovarian cancer (EOC). The expression level of ZFAS1 was detected by reverse-transcription quantitative polymerase chain reaction (RT-qPCR) assay on RNA samples extracted from different kinds of tumor tissues. The cut-off values were used to distinguish the expression of ZFAS1 by high and low levels, 4 used a median value and others did not mention. The clinical outcomes were also recorded, which included 8 studies for OS, 2 for DFS, and 2 for RFS. Furthermore, the HRs and 95% CIs were reported in 5 studies while the other 3 employed the Kaplan–Meier curves. Various clinicopathological data was reported in these studies (TNM stage in 8 studies, gender in 7 studies, metastasis in 6 studies, tumor size in 5 studies, and tumor differentiation in 3 studies).

### 3.2 Qualitative assessment

The quality of the 8 eligible studies included in the meta-analysis was assessed according to the NOS score. The quality of all articles gained more than 5 points in the methodological assessment (Table 1), indicating that these studies were of high quality.

### 3.3 LncRNA-ZFAS1 expression and OS

**Figure 2:**
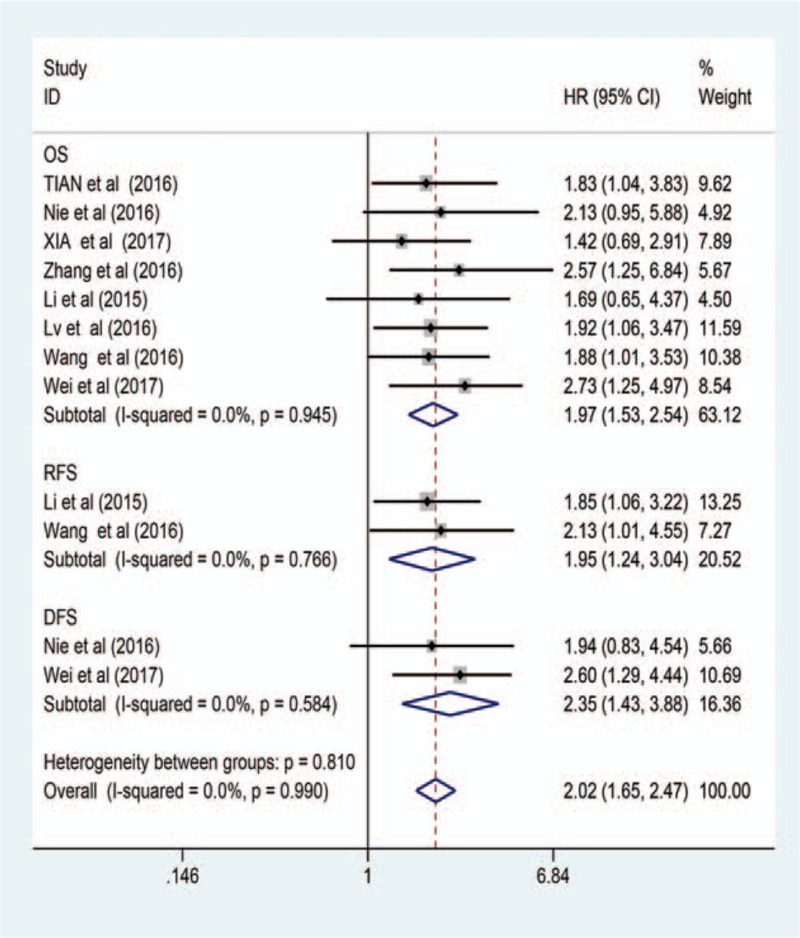
Forest plot of HR for evaluating the association between high ZFAS1 expression with OS, RFS, and DFS in cancer patients. DFS = disease-free survival, HR = hazard ratio, OS = overall survival, RFS = relapse free survival, ZFAS1 = zinc finger antisense 1.

The HR for OS was reported in all 8 studies from a total of 802 patients. A forest plot of all studies was shown in Figure 2. There was no heterogeneity among studies (*I2* = 0.0%, *Ph* = 0.946). Therefore, the fixed-effects model was used for data analysis. It was suggested that elevated ZFAS1 expression was significantly associated with poor OS (HR = 1.97, 95% CI: 1.53–2.54; Fig. 2) in various tumors. Further subgroup analysis was performed based on the cancer type, HR obtain method and sample size, although there was no heterogeneity between these articles (Table 2). Stratified analysis by HR obtain method was significantly correlated between ZFAS1 expression and OS when HR was reported (HR = 2.09, 95% CI: 1.55–2.82, *I2* = 0.0%, *Ph* = 0.884), and when data were extracted from the survival curve (HR = 1.67, 95% CI: 1.03–2.71, *I2* = 0.0%, *Ph* = 0.791). A significant relationship was observed between ZFAS1overexpression and poor OS for both digestive system cancers (HR = 2.03, 95% CI: 1.36–3.02, *I2* = 0.0%, *Ph* = 0.916) and non-digestive system cancers (HR = 1.93, 95% CI: 1.39–2.68, *I*^*2*^ = 0.0%, *Ph* = 0.637). Moreover, the upregulated expression of lncRNA-ZFAS1 was positively correlated with poor OS, including a sample size ≥100 (HR = 1.95, 95% CI: 1.35–2.81, *I*^*2*^ = 0.0%, *Ph* = 0.909) and a sample size <100 *(*HR = 1.99, 95% CI: 1.4–2.81, *I*^*2*^ = 0.0%, *Ph* = 0.639).

**Table 2:**
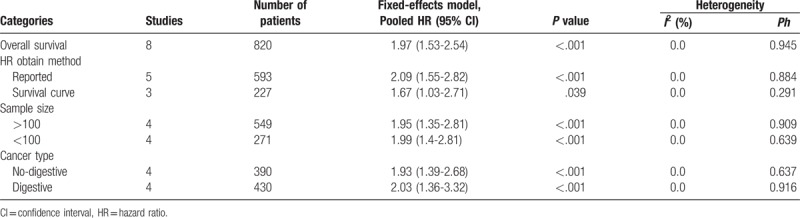
Results of the subgroup analysis for overall survival.

### 3.4 DFS and RFS

In the present meta-analysis, 2 studies that included 142 patients reported HRs for DFS. Since there was no heterogeneity (*I2* = 0.0%, *Ph* = 0.584), the fixed–effects model was applied to calculate the pooled HR; and the elevated expression level of ZFAS1 was notably correlated to poor DFS (HR = 2.35, 95% CI: 1.43–3.88). Furthermore, 2 studies that included 248 patients reported HRs for RFS, and the pooled HR revealed that there may be a significant association between the elevated expression of ZFAS1 and poor RFS (HR = 1.95, 95% CI: 1.24–3.04) with no heterogeneity (*I2* = 0.0%, *Ph* = 0.766) (Fig. 2).

### 3.5 Association of ZFAS1 expression and clinicopathological parameters

All the included studies evaluated the correlation between ZFAS1 expression and TNM stage, and the pooled ORs were 2.24 (95% CI: 1.18–4.27; *P* = .014), indicating that the overexpression of ZFAS1 was associated with advanced TNM stage. Furthermore, the relationship between ZFAS1 expression and gender or differentiation grade was also assessed. However, no significant correlation was found between ZFAS1 expression and gender or differentiation grade (*P* >.05). Five of 8 studies examined the correlation between ZFAS1 expression and tumor size, and the pooled OR of 0.71 (95% CI: −4.27; *P* = .014) suggested that the upregulated expression of ZFAS1 was associated with tumor size (size ≤5 cm vs>5 cm). In 6 studies, the association of ZFAS1 with lymph node metastasis was also investigated. It was found that the elevated expression of ZFAS1 was correlated to lymph node metastasis (pooled OR = 2.84, 95% CI = 1.53–5.27, *P* = .001). All these results were shown in Table 3.

**Table 3:**
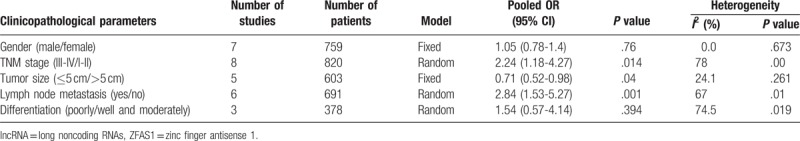
The relationship between over-expressed lncRNA-ZFAS1 and clinicopathological parameters.

### 3.6 Publication bias

**Figure 3:**
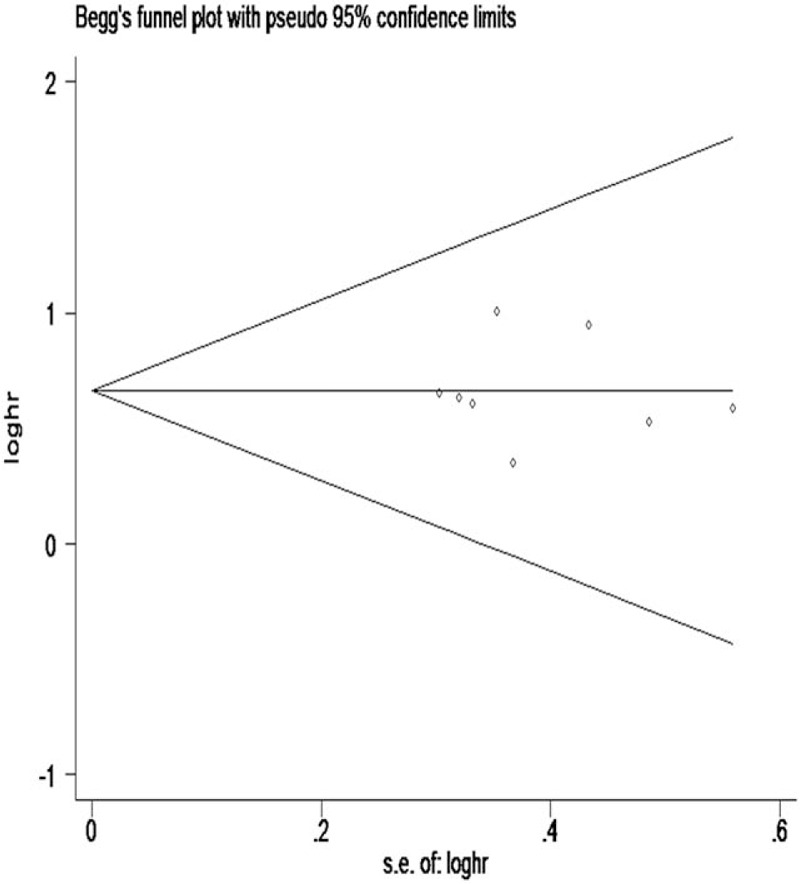
Begg's funnel plot of publication bias on the correlation between lnRNA-ZFAS1 expression and OS. OS = overall survival, ZFAS1 = zinc finger antisense 1.

**Figure 4:**
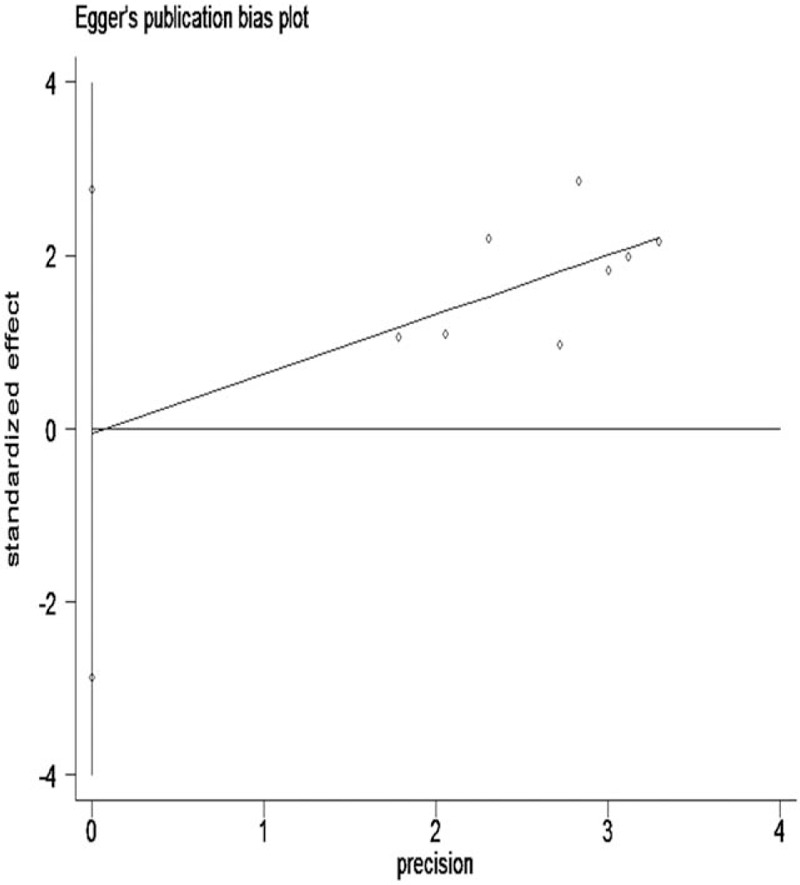
Egger's funnel plots of publication bias on the correlation between lncRNA-ZFAS1 expression and OS. lncRNA = long noncoding RNAs, OS = overall survival, ZFAS1 = zinc finger antisense 1.

In order to evaluate for publication bias of these studies, Begg funnel plot and Egger test were performed. These results revealed that there was no significant publication bias in the meta-analysis for OS (Egger test, *P* = .968; Begg test, *P* = .536) (Figs. 3 and 4). However, publication bias was not analyzed in the DFS and RFS groups due to the small number of studies.

## 4 Discussion

As a novel molecular basis, increasing evidence has shown that lncRNAs plays an important role in various human diseases, especially in cancer.^[16–20]^ The multiple roles of lncRNAs have been elucidated in various cellular biological processes such as cell proliferation, cell cycle progression, cell growth, and cell apoptosis.^[21–23]^ An increasing number of lncRNAs have been identified as targets for cancer diagnosis or potential biomarkers for cancer treatment, such as XIST, MALAT1, and H19.^[24–26]^

ZFAS1 is a recently discovered lncRNA and shown to possess an oncogenic role in tumorigenesis. Li et al reported that ZFAS1 acted as an oncogene in hepatocellular carcinoma progression by binding miR-150 and abrogating its tumor-suppressive function.^[12]^ In colorectal cancer, ZFAS1 interacted with CDK1 and was found to involve with p53-dependent cell cycle control and apoptosis in CRC cells.^[27]^ In gastric cancer, previous studies have shown that ZFAS1 promoted gastric cancer cell proliferation through the epigenetic silencing of KLF2 and NKD2 expression by binding with PRC2 and LSD1.^[11]^ In addition, Pan et al demonstrated that ZFAS1 promoted tumor growth and metastasis by inducing cell cycle progression and EMT.^[28]^ Gao et al revealed that ZFAS1 could exhibit a tumor oncogene role in glioma progression by regulating EMT and the Notch signaling pathway.^[10]^ On the contrary, several studies found that ZFAS1 may have certain tumor suppressor functions. Fan et al found that ZFAS1 overexpression significantly suppressed cell proliferation by causing cell cycle arrest and inducing apoptosis in breast cancer cells. Further functional assays indicated that ZFAS1 overexpression inhibited cell migration and invasion by regulating epithelial.^[29]^ Other studies reported that overexpression of ZNFX1-AS1 inhibited the cell proliferation and colony formation in HCC cell lines and also induced HCC cell apoptosis via regulating the methylation of miR-9.^[8]^ In breast cancer, knockdown of cytoplasmic ZFAS1 by siRNA in mouse mammary epithelial cell lines increases proliferation.^[30]^ No studies have reported that ZFAS1 exerts tumor suppressor function in other tumors by regulating protein expression. There is still no consensus regarding to the role of ZFAS1 in different tumors which imply it may work through different mechanisms.

Liu et al found that elevated ZFAS1 expression increased the risk to the OS of patients.^[31]^ Further subgroup analysis revealed a significant association between ZFAS1 and the OS of cancer patients according to sample size, tumor type, follow-up time, and analysis type. Moreover, it analyzed the relationship between ZFAS1 and pathological features, including lymph node metastasis, gender, histological grade, and TNM stage.However, through careful analysis, we found that there were still some shortcomings in these system reviews. In the present meta-analysis,^[32]^ it is noteworthy that the Kaplan–Meier plots were generated from the Cancer Genomics Browser rather than from the experiment itself in 1 included literature. Therefore, it was not correct to extract HR (95% *CI*) from the survival curves. In the clinical outcome, the systematic review denied the relationship between the expression of ZFAS1 and DFS and RFS. In addition, there was no further study on the linking ZFAS1 expression to the tumor size. Therefore, a meta-analysis was conducted to further investigate the relationship between clinicopathological features and the prognostic significance of ZFAS1 expression in various cancers.

In the present paper, we included 8 studies, 820 cancer patients and assessed the relationship between the expression level of ZFAS1 and the prognosis of tumors. Results suggested that ZFAS1 overexpression was significantly associated with shorter OS, DFS, and RFS in human solid tumors, indicating that ZFAS1 was considered as a prognostic marker for cancer patients. Analyses stratified by sample size, cancer type, HR obtain method and sample size did not alter the significant predictive value of ZFAS1 in OS from various cancers. These results suggested that the upregulation of the expression of ZFAS1 may be an independent prognostic factor for OS. In addition, the elevated expression of ZFAS1 was correlated with tumor size, TNM stage, and LNM. However, there was heterogeneity between the expression of ZFAS1 and TNM stage and lymph node metastasis. Therefore, these results need to be further demonstrated.

Certain limitations of the present study should be acknowledged. First, all studies included in this meta-analysis were conducted in China. Therefore, these results may only be representative of the Chinese population. Second, all studies enrolled in our meta-analysis were retrospective studies. The number of cancer patients and type of cancers was relatively small. Third, ZFAS1 expression was measured by RT-qPCR in all enrolled studies and the cut-off definition of high and low expression in cancer tissues was not always consistent, which may affect the results and potentially contribute to heterogeneity. Therefore, in order to make the results more accurate and perfect, different methods were used to examine the relative expression level of lncRNA ZFAS1 in cancer samples including lncRNA microarray analysis, northern blot analysis, situ hybridization, and RNA-Seq.^[33–35]^ Fourth, some studies did not provide HRs, which require the calculation or extraction HRs and 95%CIs from the available data or Kaplan–Meier curves. Fifth, the number of studies related to DFS and RFS was very limited. Hence, stratified analysis was unconditional. Finally, non-English articles or studies were not included in this analysis because negative results were not published, which could cause publication bias. Although this bias was not detected in the OS analysis. Therefore, it is possible that our results may overestimate the prognostic impact of abnormal ZFAS1 expression. Cancer is a complicated and heterogeneous disease. The role and biological functions of lncRNA ZFAS1 are different in various cancers. It is difficult to conclude the biological role of lncRNA-ZFAS1 in tumorigenesis. Heterogeneity may increase, thus affect the stability of the results. Therefore, it is necessary to conduct the larger-scale, multi-center, high-quality research to validate our results.

In conclusion, this meta-analysis revealed that the elevated expression of lncRNA ZFAS1 may be a negative factor when assessing the clinical outcome of cancer patients. Furthermore, the upregulation of lncRNA ZFAS1 might be a predictor of LNM, advanced stage, and deeper tumor invasion. These findings suggested that ZFAS1 could be used as an effective predictive biomarker for both prognosis and clinical pathology. Nevertheless, there is still necessary to conduct a better design with a larger sample to confirm our results, allowing ZFAS1 to be widely clinically applied.

## Acknowledgments

We will thank to all the authors of the included original studies.

## Author contributions

**Conceptualization:** Qing Luo, Yuanxiu Leng.

**Data curation:** Yuanxiu Leng, Xumei Chen.

**Formal analysis:** Xumei Chen, Fang Chen.

**Funding acquisition:** Qing Luo.

**Investigation:** Yuanxiu Leng, Fang Chen, Xue Wang.

**Project administration:** Qing Luo.

**Software:** Xue Wang.

**Writing – original draft:** Yuanxiu Leng.

**Writing – review & editing:** Qing Luo.
